# Acoustic Emission Source Characterisation during Fatigue Crack Growth in Al 2024-T3 Specimens

**DOI:** 10.3390/s22228796

**Published:** 2022-11-14

**Authors:** Xinyue Yao, Benjamin Steven Vien, Chris Davies, Wing Kong Chiu

**Affiliations:** Department of Mechanical and Aerospace Engineering, Monash University, Wellington Rd, Clayton, VIC 3800, Australia

**Keywords:** acoustic emission, source characterisation, fast Fourier transform, structural health monitoring, fatigue crack

## Abstract

While acoustic emission (AE) testing can be used as a valuable technique in structural health monitoring and non-destructive testing, little research has been conducted to establish its sources, particularly in 2024-T3 aluminium alloys. The major contribution of this work is that it provides a method to obtain a better linear relationship of count rate with crack growth rate based on waveform. This paper aims to characterise AE sources by synchronising the AE waveforms with load levels and then to propose possible dominant frequency ranges. The AE waveforms during fatigue crack growth in edge-notched 2024-T3 aluminium specimens, from an initial crack length of 10 mm to 70 mm, were collected at two different load ratios *R* = 0.125 and 0.5. At the same time, the crack growth rate was determined using thermal imaging and associated control software. The AE waveforms obtained were processed using the fast Fourier transform. It was shown that a significantly higher AE count rate was recorded at *R* = 0.125 compared to *R* = 0.5 when the maximum load was kept the same. This means that the *R*-ratio would affect the total amount of AE activities collected. It was also found that the dominant frequency range of the AE waveforms directly related to crack growth was 152–487 kHz, and the ranges due to crack closure were likely to be 310 kHz–316 kHz and 500–700 kHz. Based on the proposed frequency ranges, waveform selection was conducted and a better linear relationship between count rate and crack growth rate was observed. This study provides a better understanding of the AE sources and waveforms for future structural health monitoring applications.

## 1. Introduction

The health of aircraft is the basis for future space exploration and technologies. For aircraft and space vehicles, aluminium accounts for more than half of the airframe weight. Al2024 is one of the most common used alloys in aircraft components because of its high-strength and fatigue resistance [1]. Structural health monitoring of damage-tolerant aircraft components made of Al2024 is essential in determining a proper inspection interval. In particular, acoustic emission (AE) testing has been used over decades in monitoring the health of various materials. AE is defined as the method that monitors transient waves emitted from the structure when energy is released from localised sources. For metallic materials commonly used in aircrafts such as steel and aluminium, AE research has been focused on two aspects. Previous research mostly studied the hit-related feature including the rise time, count number, and energy [2,3,4]; more recent study focused on waveform-related features as it was believed that more information about sources can be revealed by them [5,6]. New algorithms or data processing methods have also been proposed for AE data [7,8]. Current applications of AE are generally qualitative; recent research aims to enable a more quantitative interpretation of AE, primarily through better source characterisation.

**Figure 1:**
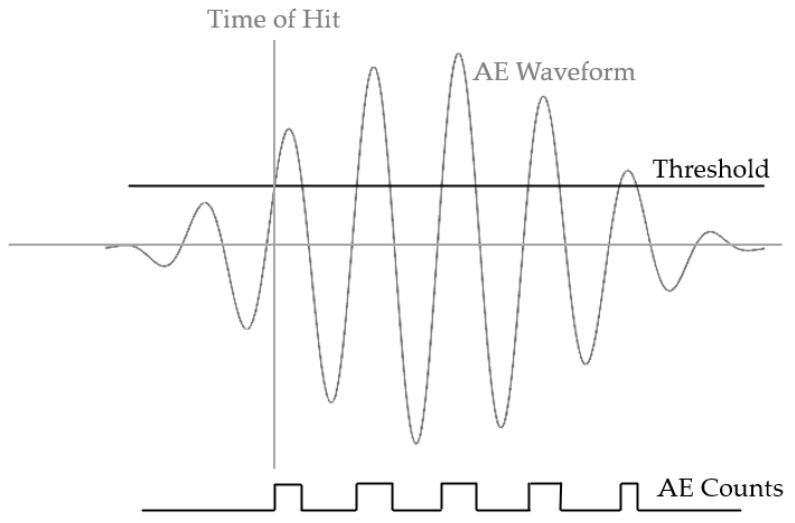
Definition of AE count.

AE count has been studied since 1983. AE count is defined as the number of times that the AE waveform excurses over the pre-defined threshold [9] as shown in Figure 1, and AE count rate is the count per unit of time. Scala and Cousland [10] found that under their experimental regime, AE occurred only at the tops of load cycles. In addition, when the crack growth rate was higher, it was found that the AE count rate was higher. Based on the comparison of the count rate of aluminium 2024 and 2124, they concluded that the AE sources for these hits that occurred at the tops of load cycles were inclusion fractures. In their study, they pointed out that the AE activity varied significantly due to the inclusion size, so the count rate needed to be averaged to observe the linear relationship between the count rate and crack growth rate. The area of inclusion for Al2024 varied from 1 to 300 μm^2^ and the majority was within the 1–25 μm^2^ range on the plane normal to the rolling plane. The minimum crack growth rate in their study was 0.1 μm per load. Other studies have also verified the relationship between crack growth rate and the AE count rate/energy rate with stress intensity range [3,11]. However, it was also found that with a different *R*-ratio, the coefficients of this linear relationship were different even with similar crack growth rate [11]. The study of Gagar et al. [12] used the crack-closure based AE results to predict the length of the crack. However, this method was geometry-based and might be hard to be extended to general usage. Other studies used the acoustic emission entropy as the parameter in monitoring the damage progress [13,14].

Recent studies focused on the waveform features including the frequency content of waveforms, the waveform patterns or wave modes, and related these features to AE sources [15,16,17]. Aggelis et al. [18] studied the duration, rise time and the ratio of rise time to the amplitude of the waveforms. They found that the change of these parameters of AE waveforms were sensitive to the fatigue damage stage. They concluded that the reason might be due to the transformation from the tensile fracture mode to the shear fracture mode. There are two types of AE sources [12] as follows: primary sources that are directly related to the crack progression, including particle fracture, crack extension, or plasticity around the crack tip; secondary sources that are not directly related to the progression of the crack, including the source from crack closure, lapping and rubbing of the crack surfaces or other activities that are due to the contact of the crack surfaces [19,20,21,22]. Bhuiyan and Giurgiutiu [5] claimed that only studying the hit-related information may not be sufficient, and they grouped the AE waveforms based on the time-domain signal and frequency spectra. In their study, nine groups of AE hits were found, among which they found that Group A, B, and D that occurred at higher load levels with higher frequency contents are responsible for crack extensions. They also found that hits that occurred when the load was low could be due to the “catching” of the saw-tooth shape of the crack surface. The differences between the current study and Bhuiyan and Giurgiutiu’s study [5] include the following: (1) there were no hits collected during the unloading process in [5], but there were hits that occurred during the unloading process in the current study; (2) the crack in [5] had two crack tips, while this study used edge-notched specimens with only one crack tip; (3) the specimen used in [5] was 1 mm thick but 3 mm thick in the current study; (4) the current study recorded AE activities for a longer range of crack from 10 mm to 70 mm; (5) the current study grouped the AE hits according to crack opening/closure. Other studies also investigated the peak frequency of hits and tried to relate them to AE sources [21,22,23,24,25,26]. The connection between the peak frequency of hits and source are summarised in Table 1 below. 

Theoretically, when the hits that were collected during crack opening are retained and the hits during crack closure are discarded a better linear relationship between count rate and crack growth rate should be obtained as the kept waveforms are directly related to the fracturing source. In this paper, the authors presented a method for selecting the waveform based on the peak frequency of AE waveforms. To do this, the primary AE waveforms when the crack is open were kept and hits during the crack closure were discarded, so that a more obvious linear relationship between count rate and crack growth rate can be observed. The AE activities under different loading ratios were also compared in this paper. 

The significance and contributions of the current work are as follows:

- A novel method of waveform selection based on the peak frequency of AE waveforms is presented. This allows a better prediction of the fatigue crack growth. Thus, the results from this study can help make a better decision on the inspection interval of components.
- The AE data collected are divided into two groups based on crack open and closure conditions at different crack lengths, and the corresponding dominant peak frequency of each group is presented to provide a basis of the waveform selection. Understanding AE waveforms from different sources also helps with AE source characterisation.
- The methodology in the current research can be extended and applied to other metallic materials.

## 2. Materials and Methods

### 2.1. Experimental Set-Up and Equipment

**Figure 2:**
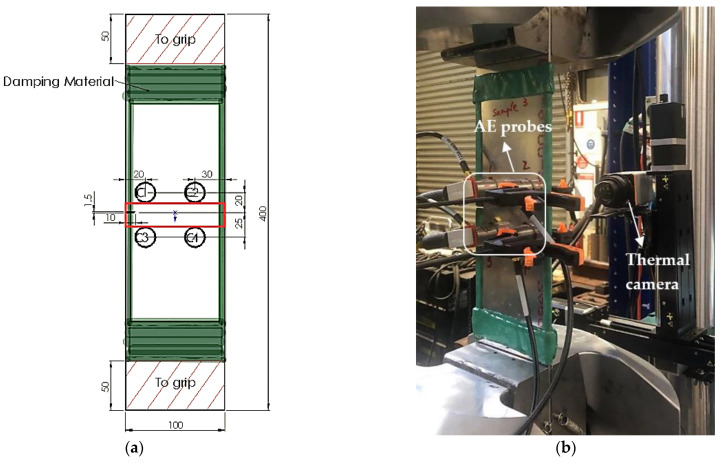

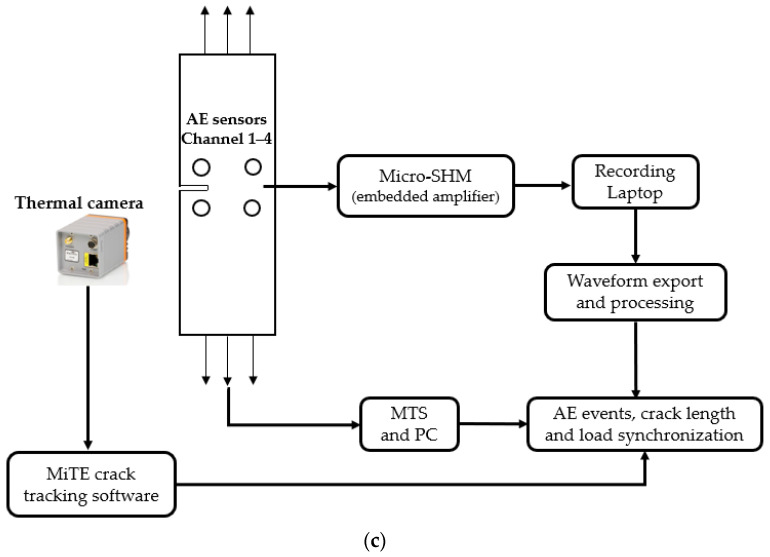
(**a**) Specimen dimensions (units in mm); (**b**) experimental set-up; (**c**) flow chart of experiments.

Four Al2024-T3 coupons of 400 mm length, 100 mm width, and 3 mm thickness as shown in Figure 2a were tested under constant amplitude sinusoidal fatigue load. A 1.5 mm wide and 10 mm long edge notch served as crack initiator. The yield strength of the material was 345 MPa. The AE recording system used was from the Physical Acoustic Corporation that consisted of a 4-channel Micro-SHM and four wideband (200–1000 kHz) PKWDI AE sensors from Physical Acoustics with built-in amplifiers. The crack tracking systems consisted of linear precision translation stages, a thermal detector, and associated control software. A longwave Gobi-640—17 μm GigE thermal camera was used along with the MiTE software [27,28] for precise crack tip monitoring with a resolution of 167 μm/pixels. During the fatigue test, the fatigue crack growth was tracked using the automated visual system. The crack tip location was recorded at a sufficiently short interval to ensure an accurate crack growth rate calculation. 

The experimental set-up is shown in Figure 2b, and a flow chart illustrating the experimental procedure is shown in Figure 2c. The face of the specimens facing the thermal camera was painted with high-emissivity black paint to aid inspection. The other face of the specimens was instrumented with an array of four AE sensors (denoted as C1 to C4) at the recording positions shown in Figure 2a. Proper grease couplant was applied between the specimen surface and the sensor surface, and the sensors were held against the specimen surface with pressure provided by lightweight clamps [9]. The specimen was applied with damping material around the edges to attenuate the noise from the grips and the reflective waves from the edges. The threshold of Micro-SHM was set to 44 dB, which is marginally above the determined noise level from acoustic recordings taken when the specimen is gripped in the tensile machine under loading.

The time when the AE waveform exceeds the set threshold level is defined as the time of hit for each waveform in the AE associated software. The waveforms were then synchronised to AE events with the AE software internal function by setting a maximum allowable time difference of arrival among all the channels, so that only hits arising from sources within the red bracketed area in Figure 2a were retained for post-processing. 

The four specimens (denoted as A, B, C, and D) were tested using an MTS machine Model 810. Each specimen was tested until the final crack length reached approximately 70 mm. Specimens A and B were tested under a 4 Hz cyclically sinusoidal far-field stress range of 6.7–53.3 MPa, corresponding to a stress ratio *R* = 0.125. Specimens C and D were first loaded under the same regime until the crack propagated for 5 mm, then the fatigue experiments were continued with the *R*-ratio increased to 0.5 by changing the minimum far-field stress to 26.7 MPa. Applying two different *R*-ratios enabled the authors to compare AE activities under different setups.

### 2.2. Data Processing

#### 2.2.1. Processing of AE Waveforms

**Figure 3:**
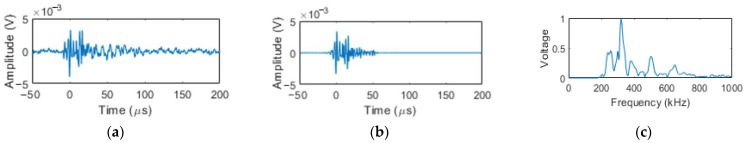
Example of: (**a**) a raw waveform; (**b**) a waveform after processing; (**c**) FFT of the waveform after processing.

To study the AE waveform frequency feature, filtering, windowing, and deconvolution were applied to the waveforms collected. The waveforms were first passed through a digital 8th order Butterworth band-pass filter where only frequency responses between 200 and 1000 kHz were kept, corresponding to the operating range of AE sensors. Then the waveforms were time-gated using a Tukey (tapered cosine) window and then processed by fast Fourier transform (FFT). Finally, the magnitudes of FFT were deconvoluted in the frequency domain with respect to the sensor calibration chart provided by the manufacturer. After filtering, windowing, and deconvolution, the correct FFT dominant frequency of waveforms could be obtained. Example waveforms before and after processing with their FFT results are shown in Figure 3. The peak frequency of waveform in the FFT result was used in selecting waveforms.

#### 2.2.2. Synchronisation of AE Hits with Load

Synchronisation of AE hits with respect to the fatigue load loading cycle helps associate the onset of the AE during the loading cycle. The fatigue tests were conducted at a nominal frequency of 4 Hz. At certain pre-determined crack lengths, synchronisation tests were performed with the loading frequency set at 0.1 Hz (i.e., 10-s period). The frequency of fatigue load was deliberately set low to enhance the confidence in associating the onset of AE with the load level. Synchronising the AE event with the phase of each fatigue loading cycle was realised by synchronising the time of the AE recording system and the time of the MTS tensile machine. The error during manual operation is estimated at less than 1 s, leading to an error of synchronisation within 10%. The specimens were loaded with 0.1 Hz frequency for approximately 500 cycles to study the synchronised AE hits as a function of the phase of the fatigue loading cycle. These synchronisation experiments were conducted at crack lengths of ~15 mm, ~30 mm, and ~70 mm. The *R*-ratio for the low-frequency test was kept at 0.125 for Specimens A and B, while, for Specimens C and D, the test of the crack length ~15 mm was with *R*-ratio 0.125, and the *R*-ratio was changed to 0.5 for the latter two lengths.

It is known that, under fatigue loading, components experience crack closure and opening. The effect of crack closure and opening can be evaluated by the *U*-ratio using the following form [29]:

(1) $$\Delta K=K_{max}-K_{min},$$

(2) $$\Delta K_{eff}=K_{max}-K_{op},$$

(3) $$U=\frac{\Delta K_{eff}}{\Delta K},$$

where ΔK is the stress intensity factor range, and $K_{min}$ and $K_{max}$ are the minimum and maximum stress intensity factors respectively; $\Delta K_{eff}$ is the effective stress intensity factor range, and $K_{op}$ is the crack opening stress intensity factor. 

The study from Newman [30] shows that the crack open stress $\sigma_{o}$ depends on $\sigma_{max}$ and the *R*-ratio as well as plane stress/strain conditions. When *R* is bigger than 0:

(4) $$\frac{\sigma_{op}}{\sigma_{max}}=A_{0}+A_{1}R+A_{2}R^{2}+A_{3}R^{3},$$

when *R* is between −1 and 0:

(5) $$\frac{\sigma_{op}}{\sigma_{max}}=A_{0}+A_{1}R,$$

when $\sigma_{op}$ is bigger than $\sigma_{min}$, the coefficients are defined as:

(6) A0=(0.825−0.34α+0.05α2)[cos(πσmax2σ0)]1α,

(7) $$A_{1}=\left(0.415-0.071\alpha \right)\frac{\sigma_{max}}{\sigma_{0}},$$

(8) $$A_{2}=1-A_{0}-A_{1}-A_{3},$$

(9) $$A_{3}=2A_{0}+A_{1}-1,$$

in which $\sigma_{0}$ is defined as the flow stress and is half the sum of the uniaxial yield stress and uniaxial ultimate tensile strength of the material. The coefficient *α* is 1 for plane stress conditions and 3 for plane strain conditions. $\sigma_{max}$ was calculated using $F_{max}/\left[specimen\;thickness*\left(specimen\;width-crack\;length\right)\right]$.

From the above equation, *U* can be calculated using:

(10) $$U=\frac{\Delta K_{eff}}{\Delta K}=\frac{K_{max}-K_{op}}{K_{max}-K_{min}}=\frac{1-\frac{K_{op}}{K_{max}}}{1-R}=\frac{1-\frac{\sigma_{op}}{\sigma_{max}}}{1-R},$$

**Figure 4:**
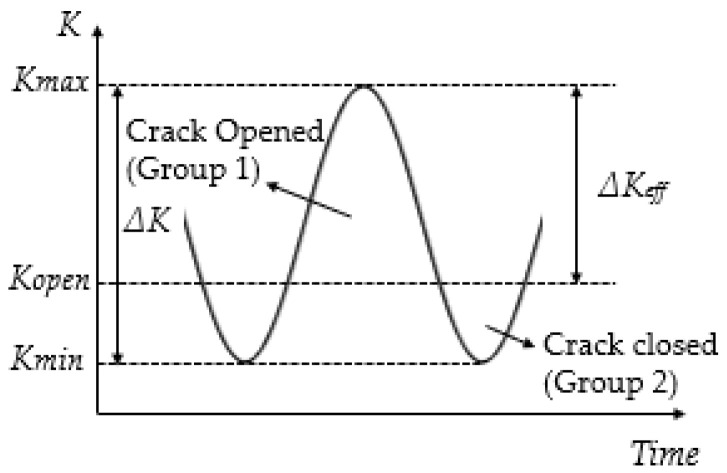
Two groups of AE hits according to crack opening and closure.

The AE hits were planned to be divided into 2 groups according to the crack opening and closure shown in Figure 4. In calculating the *U*-ratio and the load level for crack opening of the predetermined crack lengths, the coefficient *α* is needed. The value of coefficient *α* was based on the relative magnitude between the plastic zone size and the coupon thickness.

From Rice’s equations [31], the radius of the plastic zone is estimated using:

(11) $$r_{p}=\frac{1}{\pi }{\left(\frac{K_{I}}{\sigma_{ys}}\right)}^{2},$$

Thus, the radius of the plastic zone was estimated respectively for 15 mm, 30 mm, and 70 mm crack lengths. According to Newman [30], *α* = 1 when plane stress conditions exist (plastic zone size bigger than plate thickness); *α* = 3 when plane strain conditions prevail (plastic zone size smaller than 10% of the plate thickness). The value of α when the plastic zone size is between these two ranges was not specified in [30]. As the diameter of the plastic zone was greater than 10% of the plate thickness, *α* = 1 was used for Equations (6) and (7) in the current study. The calculated results of $\frac{\sigma_{op}}{\sigma_{max}}$ and *U*-ratio are shown in Table 2 below. These results are used as the standard for grouping hits at different crack lengths. In Correia et al.’s research [32], when the *R*-ratio was between 0 and 0.5, it was found that as crack length increased, the effect of crack closure was reduced. This is also accordant with the *U*-ratio calculations in Table 2. 

Hits in Group 1 hits were hits that occurred when the crack was open and when the load was relatively high, so these hits were speculated to be related to the crack extension or associated activities around the crack tip area such as inclusion fracture [10]. Group 2 hits were hits that occurred when the crack was closed. During this time, the load was too small for the crack to propagate [5], so these hits were likely to be related to crack closure where the contacting or rubbing of crack surfaces might occur. When the *U*-ratio was low, AE activities were expected to be a combination of activities associated with crack growth and crack closure. At *R* = 0.5, the crack closure was reduced as the *U*-ratio was higher compared to when *R* = 0.125, therefore the acoustic signature was expected to be more dominated by crack extension.

After the grouping, the waveforms of hits were processed with the method in Section 2.2.1 to find the frequency peak of each group, thus helping to identify the different peak frequencies of each group. 

## 3. Results and Discussion

In this section, to obtain the fatigue crack length and crack growth rate, the results from the thermal imaging equipment were analysed. After that, to observe an overall picture of the number of AE events during fatigue crack growth, AE count rate features at loading ratios *R* = 0.125 and 0.5 are shown as a function of the crack growth rate. Then the focus of the discussion turns to the waveform-related features, i.e., the frequency content of grouped AE waveforms. In Section 3.2, the synchronisation and grouping results of hits during loading frequency 0.1 Hz at certain crack lengths are discussed. In Section 3.3, the AE waveforms were selected based on their peak FFT frequency to obtain a better linear relationship between AE count rate and fatigue crack growth rate. 

### 3.1. Fatigue Crack Growth and Count Rate

**Figure 5:**
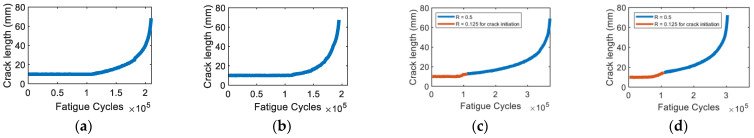
Crack growth curves: (**a**) Specimen A (*R* = 0.125); (**b**) Specimen B (*R* = 0.125); (**c**) Specimen C (*R* = 0.5); (**d**) Specimen D (*R* = 0.5).

**Figure 6:**
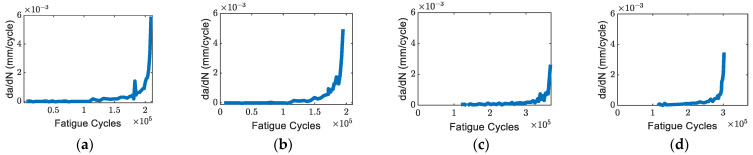
Crack growth rate: (**a**) Specimen A (*R* = 0.125); (**b**) Specimen B (*R* = 0.125); (**c**) Specimen C (*R* = 0.5); and (**d**) Specimen D (*R* = 0.5).

**Figure 7:**
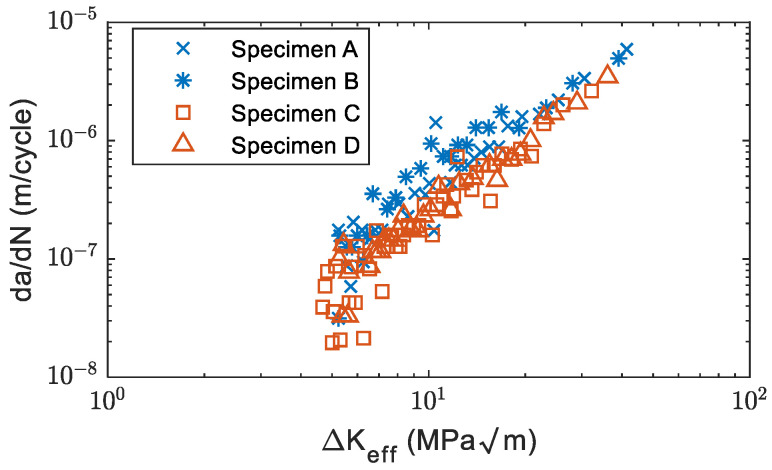
Crack growth rate versus effective stress intensity factor.

The crack propagation and crack growth rate curves of specimens obtained from the visual tracking system are shown in Figure 5a–d. As shown in Figure 5c,d, Specimens C and D were initially tested with *R* = 0.125 and then increased to *R* = 0.5 with a fixed maximum load at a crack length of around 15 mm. To reach a similar crack length, it took more fatigue cycles for Specimens C and D compared to Specimens A and B. Figure 6a–d shows the crack growth rate of four specimens. The crack growth rate versus the effective stress intensity factor is show in Figure 7. It was found that the effective stress intensity ranges at the end of Specimens A and B were slightly higher than Specimens C and D. The data in Figure 7 shows relatively good agreement with the data in the Damage Tolerance Handbook [33]. 

**Figure 8:**
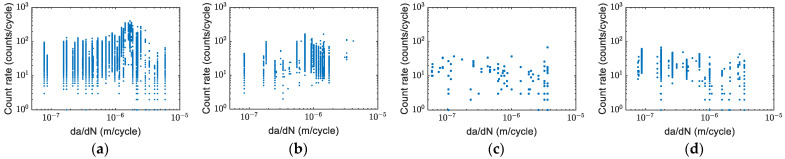
AE counts per cycle vs. crack growth rate: (**a**) Specimen A; (**b**) Specimen B; (**c**) Specimen C (after crack initiation); and (**d**) Specimen D (after crack initiation).

The count rate per cycle before averaging and the fatigue crack growth rates for specimens A, B, C, and D respectively as a function of crack growth rate (da/dN) are shown in Figure 8a–d. The count rates for Specimens A and B were generally higher than Specimens C and D. The count rate for Specimen A peaked at around da/dN ~1×10^−6^ m/cycle (Figure 8a), and the count rate for Specimen B peaked within the similar crack growth rate range (Figure 8b). The changes in count rates for Specimens C (Figure 8c) and D (Figure 8d) were not as obvious as for Specimens A and B.

In order to identify the source origin of hits that arose at different crack lengths, it is necessary to synchronise the time of hit with the load for hits under 0.1 Hz loading frequency.

### 3.2. Source Characterisation of Synchronised Hits

**Figure 9:**
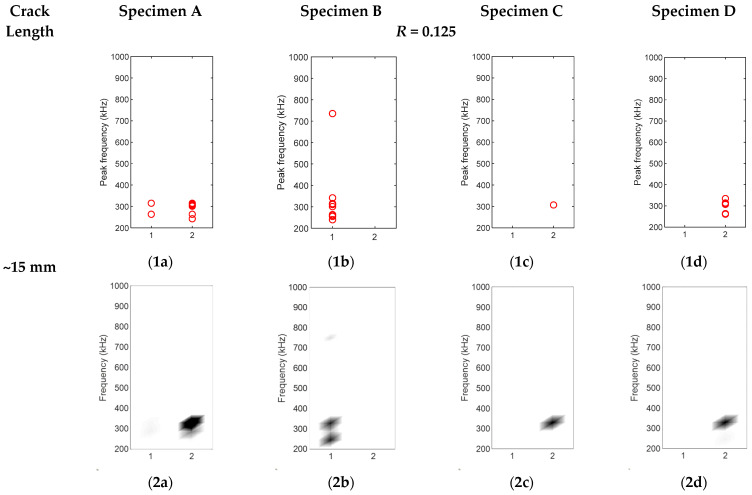
(**1a**–**1d**) Hit grouping of four specimens at crack length ~15 mm; (**2a**–**2d**) Hit grouping density of four specimens at crack length ~15 mm.

**Figure 10:**
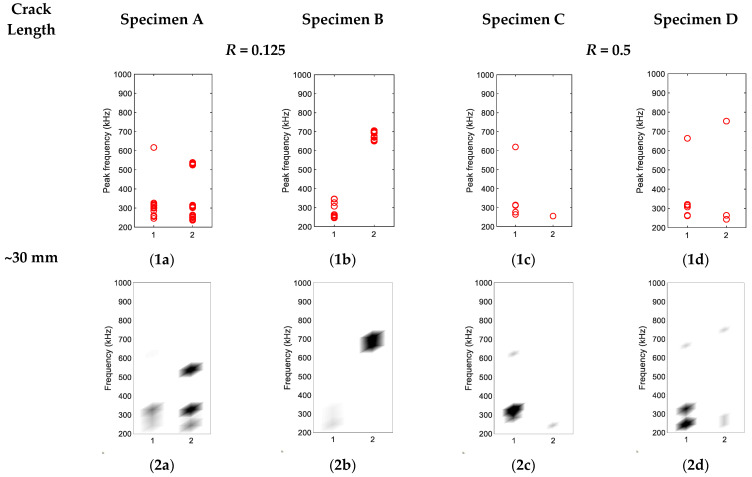
(**1a**–**1d**) Hit grouping of four specimens at crack length ~30 mm; (**2a**–**2d**) Hit grouping density of four specimens at crack length ~30 mm.

**Figure 11:**
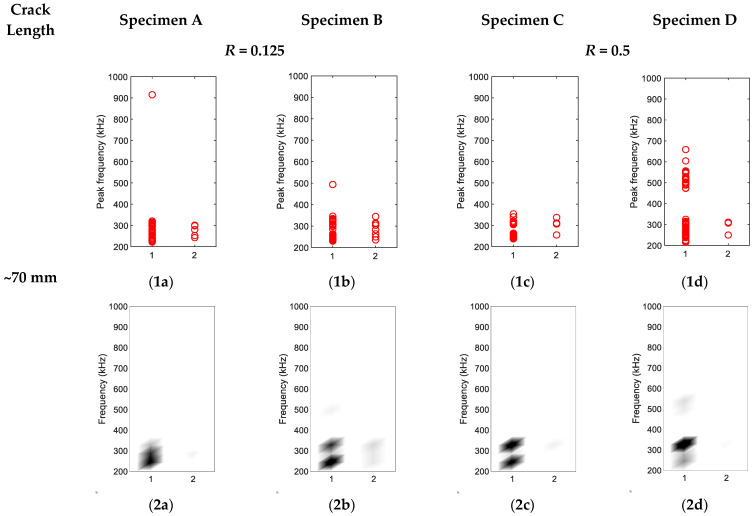
(**1a**–**1d**) Hit grouping of four specimens at crack length ~70 mm; (**2a**–**2d**) Hit grouping density of four specimens at crack length ~70 mm.

To characterise the crack closure source and crack opening source, hits collected during 0.1 Hz loading frequency were divided into two groups as described in Section 2.2.2. The groupings of hits for four specimens are shown in Figure 9, Figure 10 and Figure 11. The *x*-axis is the group number, and the *y*-axis is the peak FFT frequency of hit. The peak frequency of each waveform is represented as one circle in Figure 9, Figure 10 and Figure 11(1a–1d). The corresponding density diagrams for plots Figure 9, Figure 10 and Figure 11(1a–1d) were devised and plotted in Figure 9, Figure 10 and Figure 11(2a–2d), in which the darker colour represents the occurrence of a larger number of hits.

**Figure 12:**
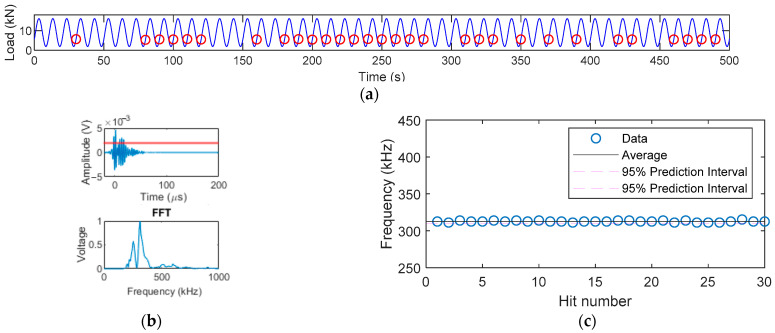
Specimen A when crack is 15 mm: (**a**) Hits during crack closure when the load was increasing; (**b**) example waveform and its FFT; (**c**) FFT peak frequency of corresponding hits.

In Figure 9, the crack had only propagated for 5 mm from the initial artificial crack. Most hits collected were grouped in Group 2 for Specimens A, C, and D. The dominant frequencies of these hits were around 310 kHz. The time of these hits respective to the load phase is shown in Figure 12a. In Figure 12a, it can be seen that these hits occurred repeatedly during low load when the load was increasing. As this load was too low for crack extension, the postulated source for these hits was that the saw-tooth shape crack surfaces might catch each other when the rough crack surfaces opened [5], leading to a group of repeated AE activities at low load. The FFT spectra of these hits are shown in Figure 12b. Their dominant frequency range was identical between 310 and 316 kHz as observed in Figure 12c.

For Specimen B in Figure 9(1b), the waveforms were collected during crack opening when the load was high. The reason for Specimen B having hits in a different group from Specimen A was due to the randomness of crack formation. Even under the exact same condition, the formed crack can be different, which probably resulted in different AE activities, and is discussed in Section 3.3.

**Figure 13:**
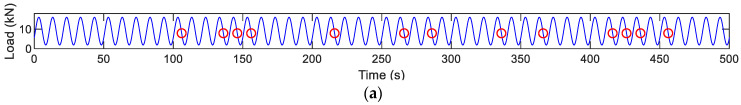

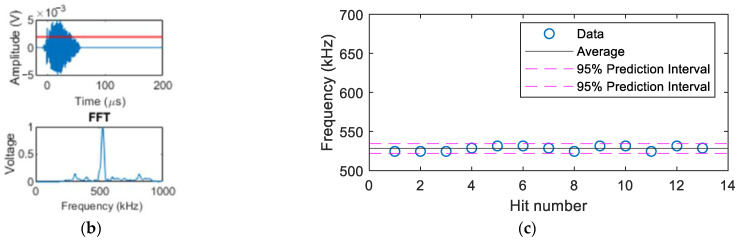
Specimen A when crack is 30 mm: (**a**) Hits during crack closure when the load was decreasing; (**b**) example waveform and its FFT; (**c**) FFT peak frequency of corresponding hits.

**Figure 14:**
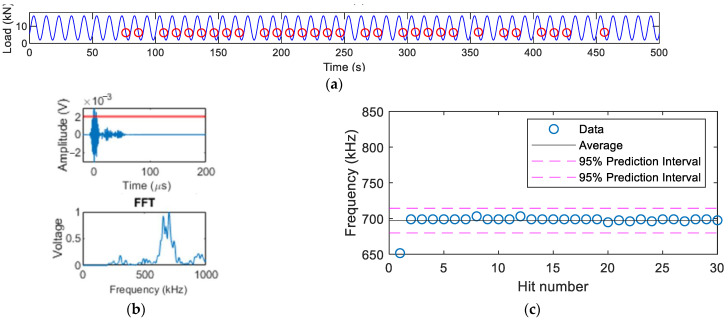
Specimen B when crack is 30 mm. (**a**) Hits during crack closure when the load was decreasing; (**b**) example waveform and its FFT; (**c**) FFT peak frequency of corresponding hits.

When the crack was 30 mm (see Figure 10), there were more high-frequency hits classified in Groups 2 for Specimens A and B. The reason for this was that the AE source caused by crack closure was intensified, resulting in contact of the crack surfaces when the crack surfaces closed during decreasing load. From Figure 13b and Figure 14b, the appearance of high-frequency content in the waveforms was also noticed. As shown in Figure 13c and Figure 14c, the dominant frequency of the AE source due to crack closure was between 500–700 kHz.

As can be seen in Figure 10(1c,2c),(1d,2d) for Specimens C and D (*R* = 0.5), even though hits classified in Group 2 did exist, the majority of the hits were still classified into Group 1. The reason could be that the *U*-ratio for Specimens C and D under *R* = 0.5 was smaller, as compared to Specimens A and B based on the U values in Table 2. The results illustrate that by keeping other conditions the same, the increase in *R*-ratio might reduce the AE activities due to the crack closure source. 

As shown in Figure 11 when the crack length was around 70 mm, only a small proportion of hits were clustered into Group 2. Most of the hits occurred during the crack opening for all four specimens and they were highly likely to be related to crack growth or activities around the crack tip, for which the dominant frequency range was in the low-frequency range.

**Figure 15:**
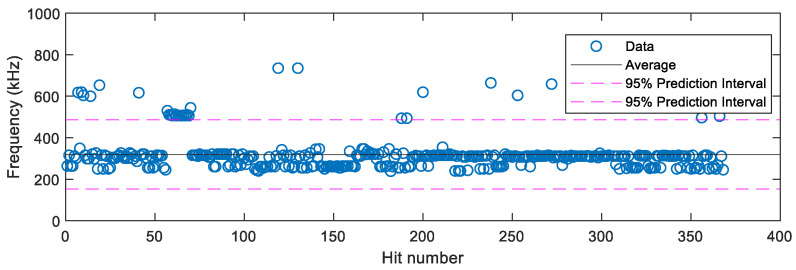
Peak frequency of hits when the crack is open.

To study the peak FFT frequency of waveforms for crack extension, the peak frequencies of hits for Specimens A, B, C, and D when the crack was open are plotted in Figure 15. Statistically, it was found that the average peak frequency of these hits was 320 kHz and that 95% of them had a peak frequency between the range of 152–487 kHz. This frequency range is similar to that seen by Bhuiyan and Giurgiutiu [5] where they found the dominant frequencies for crack extension AE sources of Al2024-T3 specimens were 100 kHz, 230 kHz, 450 kHz, and 550 kHz. 

### 3.3. The Linear Relationship between Count Rate and Crack Growth Rate

When the crack is open, the AE activities collected are expected to be dominated by crack extension or related sources such as inclusion fracture. These activities are associated directly with the extension of the crack. However, when the crack is closed, the AE sources are from contact or rubbing between the crack surfaces. These sources may be affected by the crack length, but they should not be directly related to the crack extension. Thus, discarding the hits during crack closure can potentially correct the linear relationship between count rate and crack growth rate. 

It was shown in Section 3.2 that 95% of the AE hits collected during crack opening would have a peak frequency between 152–487 kHz, and the AE hits when the crack was closed would have a peak frequency in the ranges 310–316 kHz or 500–700 kHz. In the current paper, instead of using load, the authors tried using the proposed peak frequency ranges to amend the collected AE hits. By discarding the hits that had peak frequency between the range 310–316 kHz and above 500 kHz, a relatively better linear relationship was observed. Although discarding the hits with the peak frequency range within 310–316 kHz might also result in deleting some hits during crack opening, this should not affect obtaining the linear relationship when a huge number of AE activities are considered. The reason for this is that the discarded crack-open hits could be insignificant compared to the large number of repeated hits due to closure with the dominant frequency range of 310–316 kHz. 

**Figure 16:**
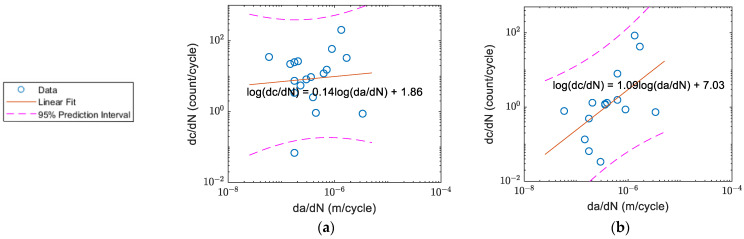
The correlation of count rate and crack growth rate for Specimen A: (**a**) before; and (**b**) after the waveform selection.

**Figure 17:**
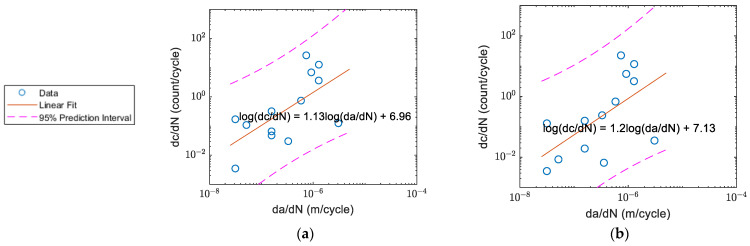
The correlation of count rate and crack growth rate for Specimen B: (**a**) before; and (**b**) after the waveform selection.

**Figure 18:**
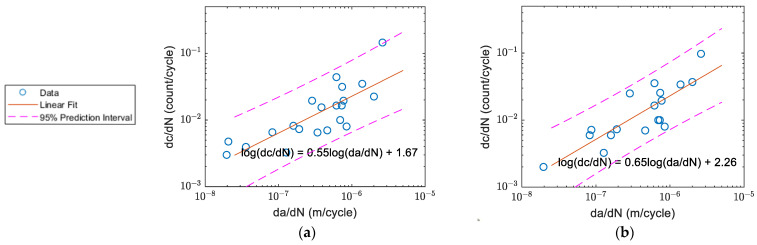
The correlation of count rate and crack growth rate for Specimen C: (**a**) before; and (**b**) after the waveform selection.

**Figure 19:**
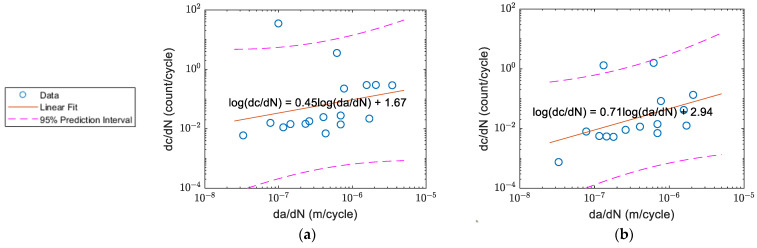
The correlation of count rate and crack growth rate for Specimen D: (**a**) before; and (**b**) after the waveform selection.

**Figure 20:**
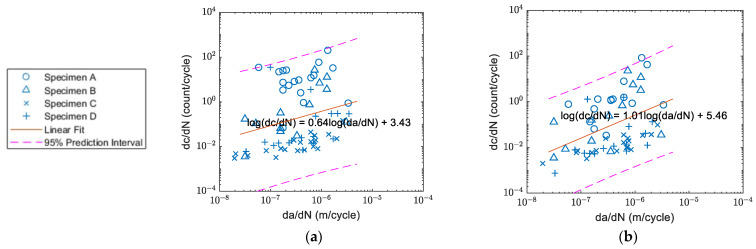
Correlation between count rate and crack growth rate: (**a**) before; and (**b**) after the waveform selection.

The correlation of count rate and crack growth of Specimens A–D before and after the waveform selection are shown in Figure 16, Figure 17, Figure 18 and Figure 19 respectively. The count rate versus crack growth rate for all specimens is shown in Figure 20. In order to obtain a reasonable plot of count rate versus crack growth rate, the count rate per cycle and crack growth rate needed to be averaged over a time interval. The area of the majority of the inclusion for Al2024 was within the 1–25 μm^2^ range [10]. To keep an increment of minimum 25 μm^2^ for the current 3 mm-thick specimens, the increment for crack growth should be at least $8.3\times 10^{-3}$ μm (~0.01 mm or 1 × 10^−8^ m). 

For Specimen A in Figure 16a, it was observed that the slope of the linear regression was only 0.14, which was distinct from the other specimens. After the waveform selection, a better correlation was observed as shown in Figure 16b with a slope coefficient close to 1. A similar trend was observed for Specimen D in Figure 19. However, this was not the same for every specimen as the activities due to crack closure were relatively random. For Specimen B in Figure 17 and Specimen C in Figure 18, the plot before and after the selection did not show obvious changes as the linear relationship could already be observed even before the selection.

Assessing from plot (b) in Figure 16, Figure 17, Figure 18 and Figure 19, it was found that after the waveform selection, the coefficient of the linear regression for Specimens C and D became closer to the coefficient of Specimens A and B. In Yu et al.’s study [11], when the crack growth rate was similar and specimen geometry was the same, the coefficients they obtained for the linear relationship between count rate and stress intensity factor range under different *R*-ratios were still different. As a result, it might be possible to have different coefficients for linear relationship between count rate and crack growth rate with different *R*-ratios. In Chai et al.’s study [26], it was also found that for a higher *R*-ratio, the energy of the AE activities was generally weaker. Their explanation was that for crack progression under a higher *R*-ratio, lower energy was required. This can also be verified from Chandran’s equation of net-section strain energy [34], where the net-section strain energy under loading regime *R* = 0.5 is lower than that under *R* = 0.125. This could be another possible reason for having different coefficients for linear regression between Specimens A and B and Specimens C and D when the same threshold was applied for them; even though, in Figure 20 where the fitting curves were obtained from data from all four specimens, a better linear relationship with a smaller scatter was observed after the selection, as the AE waveforms related to crack closure were discarded. The standard error of the linear fit was decreased by 18%. The slope coefficients and standard errors are summarised in Table 3 below. 

In this study, the AE count rate under different loading regimes was reported. It was found that by keeping the maximum load the same and increasing the minimum load, the crack closure effect could be reduced; thus, the count rate would become relatively smaller. This means that changing the *R*-ratio/*U*-ratio would also change the count rate. As a result, when monitoring the crack growth rate, the *R*-ratio should also be considered in assessing the fracture progress. After this, the AE waveforms at different crack lengths under a small loading frequency were divided into two groups according to crack open/closure. The peak frequency of each group was presented, and it was found that the peak frequency of AE waveforms also changed during crack progression. As the source characterisation is still a key problem in AE testing, example AE waveforms due to crack closure source were presented with the corresponding FFT diagrams for a deeper understanding of the AE origin. Finally, the AE waveform and peak frequency information summarised under slow loading regime was used to conduct the waveform selection for AE waveforms under 4 Hz loading frequency; a relatively better linear regression with higher sloped and smaller scattering was obtained. This result can potentially help with an improved determination of the fatigue fracture progression and thus a more appropriate inspection interval of components. The methodology from the current study can potentially be applied to other metallic materials under fatigue loading. 

One limitation of the current waveform selection methodology is that the proposed frequency range was based on hundreds of cycles under 0.1 Hz loading frequency, and it may not cover all the possible ranges, so some hits during crack closure might still not be perfectly discarded. Another limitation of this study is that only selecting the waveforms based on their peak frequency may not be entirely sufficient. More in-depth waveform selection methods need to be studied such as the waveform pattern recognition or wave mode identification of AE waveforms. Furthermore, the current study only focused on the linear regression of AE count rate and crack growth rate before and after the waveform selection. The study of other parameters that are independent of the threshold (such as the peak amplitude, the absolute energy, or the entropy) is suggested for a potentially better linear correlation with the crack growth rate for future study. 

## 4. Conclusions

This paper reported the possible AE sources of Al2024-T3 specimens during fatigue crack growth from 10 mm to 70 mm. Synchronisation of hit and load was conducted on four specimens with *R*-ratios of 0.125 and 0.5 by changing the minimum load to help distinguish between the sources of AE events from crack closure and crack formation. It was found that at a lower *R*-ratio where the *U*-ratio is higher, the crack closure source was intensified, and the general count rate was higher. This finding can enable a better experimental setup in future AE investigations, because it has been proven that the *R*-ratio can also affect the general AE count rate. It was also found that the frequency content of AE events recorded in the considered sample was dependent on the fatigue crack lengths and the *R*-ratios. It was shown that AE events directly associated with crack extension activities were likely to have a frequency content between152 kHz and 487 kHz for the considered specimens under the current experimental set-up. By contrast, AE events related to crack surface closure had a frequency content of ranges of 310–316 kHz and 500–700 kHz. This study provides a deeper understanding of the AE source characterisation, including crack closure, by in situ monitoring of the peak frequencies of AE events. The study also shows the potential of using waveform-related information in conducting waveform selection. After the selection, a more accurate monitoring of the fatigue fracture progression based on count rate can be achieved. Future studies can work on a more comprehensive selection method considering multiple selection parameters. 

## Figures and Tables

**Table 1:** Summary of frequency contents for different sources of various metallic materials.

Authors	Metallic Material	Sources	Frequency Contents
Bhuiyan and Giurgiutiu [5]	Al2024-T3	Crack extension	100 kHz, 230 kHz, 450 kHz, and 550 kHz
Joseph, Bhuiyan, and Giurgiutiu [21]	Al2024-T3	Rubbing and clapping at adjacent crack surfaces	40 kHz, 100 kHz, and 300–400 kHz
Wisner et al. [22]	Al2024-T3	Particle fracture	450–550 kHz
Pomponi and Vinogradov [23]	CuZr alloy	Plastic deformation	280–480 kHz
		Ductile crack growth Particle fracture	300–480 kHz 480–500 kHz
Han et al. [24]	AZ31 magnesium alloy	Twinning	140 kHz, 250 kHz, and 370 kHz
		Plastic events related to twinning	150 kHz, 250 kHz, and 370 kHz
		Crack extension	150 kHz
Li, Kuang, and Koh [25]	Rail steel	Crack propagation	100–350 kHz and 400–650 kHz
		Crack closure	100–350 kHz
Chai et al. [26]	316LN stainless steel	Fatigue crack growth	90–160 kHz

**Table 2:** Calculated values for *U*-ratio.

Crack Length	Specimen A and B		Specimen C and D	
	$\frac{\sigma_{op}}{\sigma_{max}}$	*U*	$\frac{\sigma_{op}}{\sigma_{max}}$	*U*
~15 mm	*R* = 0.125 (for crack initiation)			
	0.53	0.54	0.53	0.54
~30 mm	*R* = 0.125		*R* = 0.5	
	0.53	0.54	0.64	0.72
~70 mm	*R* = 0.125		*R* = 0.5	
	0.44	0.64	0.60	0.80

**Table 3:** Comparison of coefficients before and after the waveform selection.

Specimens	Before the Selection	After the Selection
A	Slope	
	0.14	1.09
B	Slope	
	1.13	1.20
C	Slope	
	0.55	0.65
D	Slope	
	0.45	0.71
A, B, C, D	Slope	
	0.64	1.01
	Standard error	
	1.38	1.13

## Data Availability

Not applicable.
